# Single nucleotide polymorphism and haplotype effects associated with somatic cell score in German Holstein cattle

**DOI:** 10.1186/1297-9686-46-35

**Published:** 2014-06-04

**Authors:** Hamdy Abdel-Shafy, Ralf H Bortfeldt, Jens Tetens, Gudrun A Brockmann

**Affiliations:** 1 Department for Crop and Animal Sciences, Humboldt-Universität zu Berlin, Berlin, Germany; 2 Department of Animal Production, Faculty of Agriculture, Cairo University, Cairo, Egypt; 3 Institute of Animal Breeding and Husbandry, Christian-Albrechts-Universität zu Kiel, Kiel, Germany

## Abstract

**Background:**

To better understand the genetic determination of udder health, we performed a genome-wide association study (GWAS) on a population of 2354 German Holstein bulls for which daughter yield deviations (DYD) for somatic cell score (SCS) were available. For this study, we used genetic information of 44 576 informative single nucleotide polymorphisms (SNPs) and 11 725 inferred haplotype blocks.

**Results:**

When accounting for the sub-structure of the analyzed population, 16 SNPs and 10 haplotypes in six genomic regions were significant at the Bonferroni threshold of *P* ≤ 1.14 × 10^-6^. The size of the identified regions ranged from 0.05 to 5.62 Mb. Genomic regions on chromosomes 5, 6, 18 and 19 coincided with known QTL affecting SCS, while additional genomic regions were found on chromosomes 13 and X. Of particular interest is the region on chromosome 6 between 85 and 88 Mb, where QTL for mastitis traits and significant SNPs for SCS in different Holstein populations coincide with our results. In all identified regions, except for the region on chromosome X, significant SNPs were present in significant haplotypes. The minor alleles of identified SNPs on chromosomes 18 and 19, and the major alleles of SNPs on chromosomes 6 and X were favorable for a lower SCS. Differences in somatic cell count (SCC) between alternative SNP alleles reached 14 000 cells/mL.

**Conclusions:**

The results support the polygenic nature of the genetic determination of SCS, confirm the importance of previously reported QTL, and provide evidence for the segregation of additional QTL for SCS in Holstein cattle. The small size of the regions identified here will facilitate the search for causal genetic variations that affect gene functions.

## Background

Mastitis is the endemic disease that causes the greatest economic losses to the dairy industry worldwide [1]. Therefore, genetic improvement through the selection of animals with a greater ability to resist or combat infection is a major breeding goal. Since a moderate to high positive genetic correlation exists between clinical mastitis and milk somatic cell count (SCC) or its logarithmic transformation (somatic cell score, SCS) [2-6], SCC and SCS have been widely used to monitor mastitis in dairy farms, although variation in SCS may be associated with different environmental conditions, different pathogens, and different physiological statuses of the animal [7]. SCS is used as an indicator trait for mastitis.

Since clinical mastitis and SCS have low heritabilities in dairy cattle *i.e.* equal to 0.10 and 0.16, respectively, traditional breeding for mastitis resistance is difficult [8,9]. Therefore, selection based on genomic information could be an interesting alternative [10]. Since genomic selection has been introduced into breeding programs, genome-wide information on SCS has been used effectively to estimate genomic breeding values for SCS. However, most SNPs (single nucleotide polymorphisms) used for genomic selection are in linkage disequilibrium (LD) with unknown causative mutations. Due to recombination between indirect markers and causative mutations, the marker effects may need to be re-estimated from time to time. Therefore, to circumvent reevaluation of SNP effects and to understand the biological mechanism behind gene variants, it is necessary to identify the causative mutations. An essential step to achieve this is the accurate mapping of genomic loci that contribute to the trait.

Compared to QTL (quantitative trait loci) studies that are performed using pedigrees, genome-wide association studies (GWAS) have the power to detect smaller chromosomal regions affecting a trait and to provide more precise estimates of the size and direction of the effects of alleles at identified loci. Recent GWAS using SNPs in US, Irish, Dutch, Scottish, and Swedish Holstein cattle identified SNPs associated with SCS on chromosomes 2, 4, 5, 6, 7, 10, 11, 12, 13, 15, 16, 18, 20, 25, 26, 28 and X [11-13].

While previous GWAS for mastitis traits in dairy cattle used SNPs, haplotype-based approaches can be more powerful for genomic regions for which allele frequencies of the tested SNP and the unknown causative mutation are different. In a population, a SNP has at most two alleles but a haplotype block can have more than two haplotypes [14]. A haplotype block consists of two or more polymorphic loci (*e.g*. SNPs) in close proximity, which tend to be inherited together with high probability. While the term allele refers to one of alternative DNA sequences at a single polymorphic locus, haplotype refers to the combination of alleles of polymorphic loci in a haplotype block on one chromosome. The combination of two haplotypes that an individual carries within a block (or homologous haplotypes) builds a diplotype, analogous to genotype for a single polymorphic locus. A haplotype can have higher LD with the allele of a QTL than individual SNP alleles that are used to construct the haplotype. Therefore, haplotypes can better separate carriers of each QTL allele and thus have larger effects than individual SNPs. Furthermore, haplotypes can also have larger effects if they combine multiple mutations on a chromosomal region that affect the trait in the same direction, which increases the power to identify genomic regions for the trait, even if they have small effects [15]. However, haplotypes can also have smaller effects if they combine QTL allele variants with effects in opposite directions. Due to the low heritability of SCS [2,9], small QTL effects are expected [16]. Thus, GWAS using haplotype information in addition to using individual SNPs could shed new light on the genetic determinants that are not captured by the single-marker approach. Therefore, the objective of this study was to identify genomic regions that contribute to differences in SCS using both SNP and haplotype information derived from genotyped SNPs.

## Methods

### Animals and phenotypes

Data were obtained from 2402 German Holstein bulls for which daughter yield deviations (DYD) for SCS were available in August 2012. The bulls were born between 1981 and 2003, with more than 97% born after 1998. Data from the National Breeding Evaluation (*Vereinigte Informationssysteme Tierhaltung (VIT)*, Verden, Germany) were available for the first three lactations. On average, each bull had 937 daughters contributing to its DYD and the mean reliability of the DYD was 88% (ranging from 72 to 99%). Estimation of DYD was based on the random regression animal model using the original daily yield records from 5 to 365 days in milk [17]. Since DYD for SCS of bulls are based on the performance data of all their daughters, adjusted for environmental effects, they are highly reliable and more accurate than the individual performance data of cows. In addition, the DYD describes the genetic value of a bull more accurately than its estimated breeding value due to the adjustment for the daughters’ dams [18].

### Genotypes and quality control

The bulls used were genotyped with the Illumina BovineSNP50 v1 BeadChip (Illumina Inc., San Diego, CA, USA), which features 54 001 SNPs across all autosomes and the X chromosome [19]. The genotyping was conducted after ethical review and approval by the committee of the GenoTrack project, reference number: 0315 134B. The SNP data from this chip were subjected to rigorous validation by the remapping procedure of Schmitt et al. [20] against the reference assembly of the bovine genome (University of Maryland bovine genome assembly, UMD3.1) [21]. This procedure mapped 53 872 oligomer sequences to a unique chromosomal position and defined 129 ambiguous SNP positions as missing due to substantial deviations between the manufacturer’s specification and the mapping strategy. During quality control, which was conducted using PLINK, release 1.07 [22], 7976 and 745 markers were excluded due to a low minor allele frequency (MAF < 0.01) and a low genotyping rate (<90% missing), respectively. Forty-eight animals were discarded because they had a high rate of missing genotypes (>10%). Furthermore, 1082 SNPs showed significant (*P* < 0.001) deviations from Hardy-Weinberg proportions and were carefully examined. Since they did not show significant associations with SCS, they were excluded from further analyses. In total, 44 576 SNPs and 2354 bulls passed the quality control. The genotyping rate of the remaining individuals was 99.3%. The subset of SNPs covered 2649.52 Mb of the bovine genome with an average distance of 59.5 kb between adjacent markers.

### Haplotype inference and block computation

Haplotypes for each chromosome were constructed using the default options in fastPHASE [23] on whole chromosomes with 10 random starts (parameter -T) and 25 iterations (parameter-C). Phased genotypes were partitioned into haplotype blocks using the solid spine algorithm implemented in the software Haploview, version 4.1 [24]. This algorithm defines a haplotype block if the first and last SNP in a region are in strong LD (D´ ≥ 0.8) with all intermediate SNPs, but the intermediate SNPs do not need to be in LD with each other. Haplotypes with a minor allele frequency below 0.01 and a genotyping error rate greater than 0.10 were excluded from further analyses. After quality control, 11 704 haplotype blocks containing 37 424 SNPs were inferred and used for GWAS. These haplotype blocks comprised 52 422 haplotypes and covered 1301.68 Mb of the genome (sum of regions between the first and last SNP in a haplotype block) with an average of three SNPs per haplotype block. The number of SNPs per haplotype block ranged from 2 to 17, with more than 95% of haplotype blocks containing two to six SNPs.

### Genome-wide association analyses

To prevent false positive associations from confounding effects, we accounted for potential population substructure using the multi-dimensional scaling (MDS) approach implemented in PLINK [22], using a pairwise population concordance (PPC) test based on an identity-by-state (IBS) similarity matrix. The MDS approach measures the similarity of alleles between independent loci (SNPs that are not in LD, *i.e.* r^2^ < 0.02) using an IBS similarity matrix across all N genotyped individuals based on the number of markers that individuals share. Then, a cluster analysis was carried out on the N*N IBS matrix [25]. The scaling process resulted in 157 significant clusters, representing axes of ancestry (*P* < 0.001). Fitting these clusters as covariates in the model for GWAS led to a reduction of the genomic inflation factor (λ) from 4.4 to 1.5. Genomic control is commonly used in GWAS to check whether spurious associations from population stratification are eliminated [26]. The idea behind this calculation is that a small number of SNPs should show a true association with a trait of interest, while the other SNPs should follow the distribution under the null hypothesis of no SNP being associated [27].

The inflation factor λ is the ratio of the observed median of the *χ*^2^ test statistic characterizing association between the phenotype and genetic markers and the expected median of this test statistic under the null hypothesis of no association predicted by theory (0.455 for 1df in association tests using the additive model). Thus, λ is a measure for the extent of the inflation of the excess of type 1 error [27]. Due to differences in allele frequencies caused by population stratification, observed values of the test statistic can be inflated above their expectations under the null hypothesis [28]. To prevent false negative associations, we included the most significant SNPs of a genome-wide scan as covariates into the model in a stepwise manner, to detect additional loci [29]. The stepwise adjustments for the most significant SNP effects led to a further reduction of λ to 1.4.

The GWAS was performed using the linear regression procedure implemented in PLINK [22], where the DYD for SCS were regressed on the number of copies of a particular allele at the SNP using the PLINK linear option, including population stratification as covariates. SNPs and haplotypes were considered significant at a genome-wide threshold of α < 0.05 after Bonferroni correction if the nominal *P-*value × K was less than or equal to 0.05, where K is the number of tests conducted in the GWAS; K = 44 576 for SNP analyses and K = 52 422 for haplotype analyses. To visualize the GWAS results, Manhattan plots of -log_10_*P-*value were generated using Haploview, version 4.1 [24]. Then, a Bonferroni test was performed to test phenotypic differences between either genotype or haplotype groups of significant SNPs or haplotype blocks, respectively, using SAS® software, version 9.2 (SAS® Institute Inc., 2008, Cary, NC, USA). To estimate the additive genetic variance explained by a single SNP or haplotype, we used the formula 2*β*^*2*^*f* (1-*f*), where *β* denotes the estimate of the allele substitution effect; *i.e*. the effect of the locus per copy of the variant allele, and *f* denotes the frequency of the variant allele. For haplotypes, the ‘allele substitution’ effect depends on the haplotype that is set to zero. Briefly, the genetic variance calculated by this method determines the contribution of the SNP or haplotype to the additive genetic variance based on its estimated effect and haplotype/allele frequency under Hardy-Weinberg equilibrium and an additive polygenic model [30].

### Selection of candidate genes

In several dairy cattle breeds, non-zero levels of LD (r^2^ ≥ 0.06) among markers were reported to extend up to 1 Mb [31]. Therefore, we used 5′ and 3′ flanking regions of 1 Mb around a significant SNP or up- and downstream from a significant haplotype block to search for candidate genes, which could be responsible for the observed significant associations with SCS. The start and end positions of genes were extracted from the Ensembl database (UMD3.1 Ensembl data base build 73, http://www.ensembl.org).

SNPs and haplotype blocks were assigned to genes using an Ensembl Perl API tool (http://www.ensembl.org) through a homemade Perl script (http://www.perl.org) to identify all possible genes within the flanking regions that could be in LD with the causative mutation. Gene ontology analysis was performed using a Perl script (http://www.perl.org) to extract the functional annotation derived from UniProtKB/Swiss (http://www.uniprot.org/uniprot) and GeneCards (http://www.genecards.org). A gene was selected as a candidate if the gene ontology annotations associated with the gene included immune-related functions.

## Results

**Figure 1:**
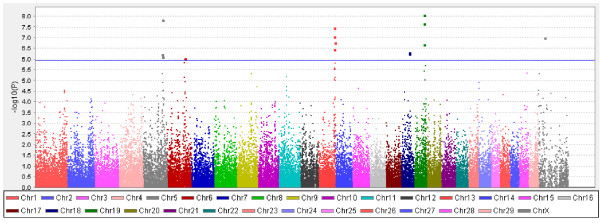
**Genome-wide association analysis for DYD of SCS in German Holstein cattle.** The Manhattan plot demonstrates the results of association after correction for population structure; the horizontal blue line indicates the whole-genome significance threshold after Bonferroni correction at α = 0.05 (*P* ≤ 1.14 × 10^-6^).

**Figure 2:**
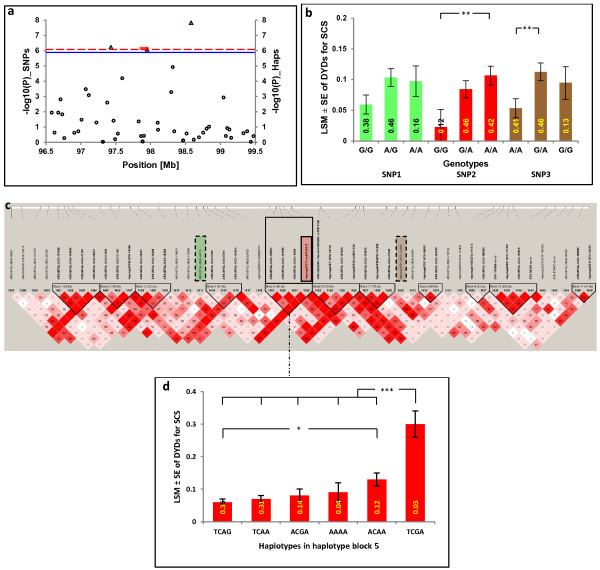
**Significant region on BTA5 associated with DYDs for SCS. (a)** Manhattan plot for GWAS of significant SNPs and haplotypes; horizontal blue and red dashed lines indicate the whole-genome significance thresholds at *P* ≤ 0.05 after Bonferroni correction for single markers and haplotypes, respectively; triangles refer to significant SNPs and bars refer to significant haplotypes. **(b)** Genotype effect plot of the three significantly associated SNPs. **(c)** Linkage disequilibrium (LD) and haplotype block structure of the significant region on BTA5. Each box represents the LD, measured by D′, corresponding to each pair-wise SNP; haplotype blocks are indicated with black triangles, significant SNPs are highlighted in color and significant haplotypes are framed. **(d)** Haplotype effect plot of significantly associated haplotypes. *(*P* < 0.05), **(*P* < 0.01), and ***(*P* < 0.001) indicate significant differences among groups. Numbers inside the columns of **(b)** and **(d)** indicate genotype and haplotype frequencies; **SNP1 =** *ARS-BFGL-NGS-44153*; **SNP2 =** *Hapmap53773-ss46526912*; **SNP3 =** *Hapmap47511-BTA-114200*; see Additional files 1 and 2 for information on significant regions of other chromosomes.

In the genome-wide analysis of 2354 progeny tested bulls, 16 SNPs reached genome-wide significance for association with DYD for SCS (α = 0.05, *P* ≤ 1.14 × 10^-6^, Table 1 and Figure 1). Among these SNPs, three were located on BTA5 between 97.4 and 98.6 Mb, two on BTA6 between 85.5 and 88.1 Mb, five on BTA13 between 78.6 and 83.3 Mb, two on BTA18 between 43.3 and 43.4 Mb, three on BTA19 between 50.6 and 52.4 Mb, and one on BTAX at 30.6 Mb (Figure 2) and (see Additional file 1: Figures S1, S2, S3, S4 and S5). The haplotype analysis did not identify other genomic regions than those detected by single-marker analysis. Among the 10 haplotypes that were significantly associated with SCS on the five autosomes (*P* ≤ 9.8 × 10^-7^, Table 2), eight harbored significant SNPs, while the other two haplotypes were either between significant SNPs, *i.e.* on BTA6, or located very close to a significant SNP, *i.e.* on BTA13 (Figure 2) and (see Additional file 1: Figures S1, S2, S3, S4 and S5). No additional SNP was identified after the stepwise adjustments for the effect of these significant SNPs (see Additional file 2: Table S1).

**Table 1:** SNPs associated with DYD for SCS

**SNP ID**	**Chr**	**Pos (bp)**	**FA**	**FAF**	**β**	***P*-value**	**Genetic variance %**^**#**^	Nearest gene*	**Nearby immune genes**^**§**^
ARS-BFGL-NGS-44153	5	97430973	G	0.61	-0.08	5.87E-07	0.30	GPRC5A	CDKN1B
Hapmap53773-ss46526912	5	97948752	G	0.35	-0.09	8.00E-07	0.37	MANSC1	CDKN1B
Hapmap47511-BTA-114200	5	98579869	A	0.64	-0.09	1.47E-08	0.37	ETV6	
BTA-77077-no-rs	6	85527109	A	0.60	-0.09	1.39E-06	0.39	TMPRSS11F	TMPRSS11D
ARS-BFGL-NGS-112872	6	88069548	A	0.68	-0.10	9.68E-07	0.44	DCK	DCK, IGJ, DBP
ARS-BFGL-NGS-95538	13	78644697	G	0.86	-0.12	3.55E-08	0.35	SLC9A8	UB2V1
Hapmap47255-BTA-34035	13	79730805	A	0.67	-0.08	8.73E-08	0.28	KCNG1	NFATC2
BTA-33950-no-rs	13	80094921	A	0.42	-0.08	3.67E-07	0.31	NFATC2	NFATC2
Hapmap32551-BTA-128831	13	81743652	A	0.74	-0.08	1.68E-07	0.25	snoU2_19	
ARS-BFGL-NGS-14974	13	83242122	A	0.90	-0.12	1.50E-06	0.26	DOK5	
Hapmap52325-rs29020544	18	43327273	C	0.43	-0.07	4.86E-07	0.24	RGS9BP	CEBPG
ARS-BFGL-NGS-57076	18	43379174	A	0.42	-0.07	5.85E-07	0.24	TDRD12	CEBPG
Hapmap57515-ss46526957	19	50647677	A	0.19	-0.12	2.19E-08	0.44	FOXK2	FOXK2, CD7, NPB
ARS-BFGL-NGS-117290	19	51680150	A	0.37	-0.08	2.15E-07	0.30	GCGR	NPB, HGS
UA-IFASA-5300	19	52436005	A	0.30	-0.10	9.04E-09	0.42	RPTOR	CARD14
Hapmap47243-BTA-31267	X	30639394	A	0.79	-0.11	1.04E-07	0.40	U6	

Chr = chromosome; FA = favorable allele; FAF = favorable allele frequency; β = change per favorable allele (regression coefficient); ^#^genetic variance for favorable alleles is based on the formula *2β2f (1-f)*, where *β* denotes the change per favorable allele and *f* denotes the frequency of the variant; *nearest gene = genes within an area of 1 Mb centered at the SNP; ^§^nearby immune genes = genes of known immune functions within a window of 1 Mb around SNP; positions are according to the University of Maryland bovine genome assembly (UMD3.1); candidate genes are extracted from the Ensembl data base (UMD3.1 Ensembl data base build 73).

**Table 2:** Haplotypes associated with DYD for SCS

**Hap ID**	**Position (bp)**			**Length (Mb)**	**HapA**	**HF**	**β**	***P*-value**	**Genetic variance %**^**#**^	**Genes within HTB**	**Other nearby genes**^**‡**^
	**Chr**	**Start**	**End**								
BTA5_Hap5*	5	97862816	97948752	0.09	TCAG	0.35	-0.09	7.05E-07	0.37	LOH12CR1, MANSC1	*CDKN1B*
BTA6_Hap4*	6	85142067	85527109	0.39	CAGG	0.34	0.09	1.84E-07	0.44	GNRHR, *TMPRSS11D*	UBA6
BTA6_Hap8	6	86642355	86904512	0.26	AGGG	0.38	0.107	4.08E-08	0.54	LOC781988, UGT2A1	SULT1B1
BTA6_Hap12*	6	87929420	88174863	0.25	AAGGGC	0.32	0.103	3.37E-07	0.46	GRSF1, MOB1B, *DCK*	*IGJ*, *DBP*
BTA13_Hap10*	13	78383148	78858126	0.47	AAAGGAG	0.14	0.12	3.60E-08	0.33	SLC9A8, RNF114	*UB2V1*, *PTGIS*
BTA13_Hap17*	13	79730805	79775537	0.04	GA	0.34	0.08	9.79E-08	0.30		KCNG1, *NFATC2*
BTA13_Hap34	13	83917405	84002346	0.08	ACAA	0.12	0.12	3.94E-08	0.31		CBLN4
BTA18_Hap6a*†	18	43327273	43379174	0.05	CA	0.41	-0.07	4.61E-07	0.27	RGS9BP, TDRD12	*CEBPG*
BTA18_Hap6b*†	18	43327273	43379174	0.05	AG	0.58	0.07	7.38E-07	0.26	RGS9BP, TDRD12	*CEBPG*
BTA19_Hap7*	19	50547082	50647677	0.10	GAA	0.19	-0.11	2.64E-08	0.39	RAB40B, FN3KRP	*FOXK2*, *CD7*, *NPB*

Chr = chromosome; Hap ID = haplotype ID; Mb = megabase; HapA = haplotype alleles; HF = haplotype frequency; β = change per significant haplotype (regression coefficient); HTB = haplotype block; BTA = *Bos taurus* autosome;

^#^genetic variance for significant haplotype is based on the formula *2β2f (1-f)*, where *β* denotes the change per significant haplotype and *f* denotes the frequency of the variant; ^‡^other nearby genes = genes within an area of 1 Mb upstream or downstream of the haplotype block boundaries; genes of known immune functions are written in italic; ^*^haplotype blocks contain SNPs which are significant in GWAS for single marker analyses; † two significant haplotypes were identified in this haplotype block, BTA18_Hap6 (a for the first haplotype and b for the second one); positions follow the University of Maryland bovine genome assembly (UMD3.1); candidate genes are extracted from the Ensembl data base (UMD3.1 Ensembl data base build 73).

The genetic variance explained by all significant SNPs considered together was equal to 5.4% of the total genetic variance of the analyzed population, while the estimated variance for each significant SNP separately ranged from 0.25 to 0.44% (Table 1). The genetic variance explained by all significant haplotypes was equal to 3.7% and ranged from 0.26 to 0.54% for each haplotype separately (Table 2). The sum of the estimated variances attributed to the leading SNPs for each haplotype block that contained significant haplotypes (*i.e.* the SNPs with the lowest *P*-value for a significant haplotype) accounted for 2.7% of the total genetic variance.

The most significant SNP (*P* = 9.04 × 10^-9^, effect size for the minor allele equal to -0.10 units of DYD for SCS) was located on BTA19 (*UA-IFASA-5300* at 52.44 Mb), while the SNPs with the largest favorable effect size (-0.12 units of DYD for SCS) were on BTA13 (*ARS-BFGL-NGS-95538* at 78.64 Mb, *P* = 3.55 × 10^-8^ and *ARS-BFGL-NGS-14974* at 83.24 Mb, *P* = 1.50 × 10^-6^) and BTA19 (*Hapmap57515-ss46526957* at 50.65 Mb*, P* = 2.19 × 10^-8^) (Table 1). The SNP on BTA19 (*Hapmap57515-ss46526957*) was also located in the most significant haplotype (*BTA19_Hap7, GAA,* 50.55 to 50.65 Mb, *P* = 2.64 × 10^-8^) and which had the largest favorable effect size (-0.12 units of DYD for SCS) (Table 2).

Interestingly, the most frequent SNP alleles in the identified genomic regions on BTA6 and BTAX were associated with lower DYD for SCS, which indicates lower mastitis incidence (Table 1). Nonetheless, the significant haplotypes on BTA6, which were also the most frequent (0.32 to 0.38), were associated with an increase in SCS (Table 2). In the genomic regions on BTA18 and 19, all alleles with negative effects on SCS were the minor SNP alleles; the significant haplotypes on BTA18 and 19 also had a low frequency and were associated with reductions in SCS (Tables 1 and 2).

The regions identified on BTA5 and BTA13 contained both types of alleles, highly frequent favorable and highly frequent unfavorable alleles. On BTA5, the major alleles of the two distal SNPs (*ARS-BFGL-NGS-44153* and *Hapmap47511-BTA-114200*), with frequencies of 0.61 and 0.64, respectively, were associated with lower SCS, while the major allele of the third significant SNP (*Hapmap53773-ss46526912,* with a frequency of 0.65) was associated with higher SCS. The significant haplotype in this region decreased SCS and was the most frequent haplotype (0.35). Interestingly, the frequencies (0.35) and the proportion of genetic variance explained (0.37%) were the same for allele G of the SNP *Hapmap53773-ss46526912* (SNP number 4 in the haplotype block) and the haplotype TCAG of the haplotype block BTA5_Hap5 (Tables 1 and 2). On BTA13, the major alleles of four of the five significant SNPs were associated with lower DYD for SCS; an exception was the SNP in the middle, for which the minor allele (frequency equal to 0.42) was the favorable one (Table 1). All significant haplotypes of the associated haplotype block on BTA13 (frequencies between 0.12 and 0.34) were associated with high SCS (Table 2). Opposing effects of the major alleles of the SNPs in a region that are significantly associated with SCS could indicate the presence of different mutations in this region or loss of linkage with the causal mutation(s) due to historic recombination between the significant SNPs and the linked mutation.

## Discussion

Using SNP and haplotype data, we identified six genomic regions associated with DYD for SCS. The identified regions on BTA5, 6, 18 and 19 are in regions where previously reported QTL for clinical mastitis and/or SCS have been mapped by linkage analyses in structured pedigrees (See Additional file 1: Figure S6). Most interesting is the significant region on BTA6 from our GWAS, which coincided with QTL that have repeatedly been mapped for SCS in German and French Holstein cattle [32], for clinical mastitis in Danish Holstein cattle [33], and in a GWAS for SCS in US Holstein cattle [11]. The GWAS in US Holstein cattle identified three SNPs between 85.2 and 88.90 Mb on BTA6 associated with SCS that are located in the same region than that identified in our study. Although the same BovineSNP50 BeadChip was used in the US Holstein cattle study [11], different SNPs in this region were significantly associated with SCS in our study. The identified region on BTA5 is located in a QTL region for SCS that was found in US Holstein cattle [34]. The significant SNPs on BTA18 and 19 were also supported by known QTL in German and French Holstein cattle [32,35]. Our study did not identify associations in QTL regions that had been previously reported with suggestive significance in German Holstein cattle by linkage analyses, *e.g*., QTL identified on BTA2 [36], BTA7, BTA10, and BTA27 [37].

Although, many significant regions identified by GWAS [11-13] overlap with QTL from linkage studies, several regions were only identified by GWAS (see Additional file 1: Figure S6). With respect to our study, the regions on BTA13 and BTAX have not been reported before to be associated with SCS, neither in Holstein nor in other cattle breeds (http://www.animalgenome.org/cgi-bin/QTLdb/BT/index). The regions detected in our study are representative of the German Holstein population since almost all German breeding sires contributed to the analyzed bull population. Loci that were identified in other populations but not in the present analysis probably have too small effects to be detected in the German Holstein sire population, have different LD, are not segregating in the German Holstein population, or were false positives in the other studies.

An important factor for GWAS is the elimination of spurious associations that may result from relationships among individuals [28]. In the current study, we accounted for population stratification using the genomic information of every bull. Ideally, the inflation factor, λ, for genomic control should be equal to 1, which would reflect the assumption that only a small fraction of the tested loci show true associations [27]. In the current study, even after correction for population stratification effects, λ had a value of 1.5. This inflation may be explained by the polygenic nature of SCS, with a large number of contributing loci, each with a small effect, and/or by causative mutations being in LD with multiple genotyped SNPs [38-41].

Compared to linkage studies in German Holstein cattle, which provided large confidence intervals for QTL, our GWAS using the BovineSNP50 BeadChip identified much smaller chromosomal regions, with lengths ranging from 0.05 to 5.62 Mb. GWAS uses historical recombination events over many generations across the genome, including those in the interval surrounding a mutation that affects a trait. Thus, GWAS can narrow detected effects to relatively small genomic regions linked to an associated SNP in the population, in which only few genes reside [42]. In most cases, SNPs identified by GWAS are not causative mutations themselves, but merely linked to one or several causative mutations. Although the significant SNPs or haplotypes identified by GWAS may not represent the causative mutation, the identified significant intervals are much smaller than the QTL intervals that result from linkage analysis of pedigrees. For instance, the QTL on BTA6 identified in [32] (*P* = 0.04, chromosome-wise), with a peak QTL position at 99 cM (≈90 Mb) and a 95% confidence interval from 16 to 135 cM (≈14.5 to 122.7 Mb), could be reduced in our study to a 3.1 Mb interval from 85.1 and 88.2 Mb. Likewise, the QTL previously identified on BTA18 with a peak QTL position at 72.55 cM (≈46.2 Mb) and a 95% confidence interval from 64.1 to 74.6 cM (≈40.8 to 47.5 Mb) [35] was located in a 0.05 Mb interval in our study.

Of particular interest is the region on BTA6 between about 85 and 89 Mb, which has been associated with mastitis traits in several studies [11,32,33]. In our study, the region between 85.1 and 88.2 Mb was significant, which contains the following candidate genes: *TMPRSS11D* (*transmembrane protease serine 11D*), *DCK* (*deoxycytidine kinase*), *IGJ* (*immunoglobulin J chain*), and *DBP* (*vitamin D-binding protein*, also known as *GC-globulin, group-specific component*) (Tables 1 and 2). *TMPRSS11D* plays an important role in the activation of the pro-macrophage-stimulating protein [43], which induces macrophage spreading, migration, phagocytosis, and cytokine production. It also inhibits the lipopolysaccharide-induced production of inflammatory mediators [44-46]. *DCK* has a functional role for drug resistance and sensitivity [47] and *IGJ* regulates the structure and function of IgM polymers secreted by B cells [48] and helps to bind immunoglobulins with secretory components [49]. *DBP* is a multifunctional protein that associates with membrane-bound immunoglobulin on the surface of B-lymphocytes [50] and with the IgG receptor on the membranes of T-lymphocytes [51].

Although all significant associations were observed in regions for which both SNPs and haplotypes had effects, some haplotypes did not contain significant SNPs (BTA6_Hap8, and BTA13_Hap34). This is attributed to the nature of the haplotype-based methods, which can better detect functional haplotypes such as *cis*-interactions among multiple DNA variants in a haplotype block region [52,53], which is an advantage of haplotype analysis compared to the SNP analysis. Likewise, some significantly associated SNPs did not belong to a haplotype block (*ARS-BFGL-NGS-44153* and *Hapmap47511-BTA-114200* on BTA5; *UA-IFASA-5300* on BTA19), or they were located in haplotype blocks that did not show significant associations (*BTA-33950-no-rs*, *Hapmap32551-BTA-128831*, and *ARS-BFGL-NGS-14974* on BTA13; *ARS-BFGL-NGS-117290* on BTA19; *Hapmap47243-BTA-31267* on BTAX). The power for detecting association is maximized if the frequencies of a specific marker allele and a causal DNA variant are similar. LD can be high only if the two alleles (the observed marker allele and the hidden causal mutation) have a similar frequency and are located on the same chromosome. Forming haplotypes with several contiguous SNPs in a block could change the combination of SNP alleles (*i.e.* haplotypes) on a chromosome and reduce the strength of association with a causal SNP in such cases [54].

All SNPs and haplotypes associated with SCS in our study explained only a small proportion of the total genetic variance. This is due to the low heritability and the complex nature of the SCS trait, which involves the effects of a large number of variants. The effects of potential additional loci were probably too small to pass the stringent genome-wide significant threshold, or the causal variants were too far away (low LD) from the SNPs that were genotyped, or the causal variants had a different allele frequency than the genotyped SNPs (incomplete LD).

## Conclusions

This study is the first GWAS for SCS in German Holstein cattle. The results provide further evidence for previously reported QTL for SCS on BTA5, 6, 18 and 19 in Holstein cattle, which were fine-mapped in our GWAS. In addition to known QTL, we identified QTL on BTA13 and the X-chromosome that have not been reported before. In the comparison of GWAS using SNPs versus haplotype, our results demonstrate that GWAS using haplotypes provides some information that was not obtained by SNP analyses alone. Thus, GWAS using SNP and haplotype information can contribute to increase the proportion of genetic variance explained by QTL. Although SNP chips with higher density and next-generation sequencing may provide new data in the near future, the results of our study suggest that the 50 k bovine BeadChip is a valuable source of information to discover mechanisms that contribute to high and low SCS or to different susceptibilities for mastitis.

## Abbreviations

BTA: *Bos taurus* autosome; BTAX: *Bos taurus* chromosome X; cM: Centimorgan; DYD: Daughter yield deviations; GWAS: Genome-wide association study; IBS: Identity-by-state; LD: Linkage disequilibrium; MAF: Minor allele frequency; Mb: Mega base; MDS: Multi-dimensional scaling; PPC: Pairwise population concordance; QTL: Quantitative trait loci; SCC: Somatic cell count; SCS: Somatic cell score; SNP: Single nucleotide polymorphism; VIT: Vereinigte Informationssysteme Tierhaltung; λ: Genomic inflation factor.

## Competing interests

The authors declare that they have no competing interests.

## Authors’ contributions

HA performed the analyses and wrote the manuscript. RHB prepared and tested the script files. JT organized SNP genotyping. GAB designed the study and contributed to writing the manuscript. All authors read and approved this manuscript.

## Supplementary Material

Additional file 1: Figures S1, 2, 3, 4, and 5 Significant regions on BTA6, 13, 18, 19 and X associated with DYD for SCS, respectively. **(a)** Manhattan plots for GWAS of significant SNPs and haplotypes; horizontal blue and red dashed lines indicate whole-genome significance thresholds at *P* ≤ 0.05 after Bonferroni correction for single markers and haplotypes, respectively; triangles refer to significant SNPs and bars refer to significant haplotypes. **(b)** Genotype effect plots of the significantly associated SNPs. **(c)** LD and haplotype block structure of the significant regions; each box represents the D′ values corresponding to each pair-wise SNP; haplotype blocks are indicated with black triangles, significant SNPs are highlighted in color and significant haplotypes are framed. **(d)** Haplotype effect plots of significantly associated haplotypes; *(*P* < 0.05), **(*P* < 0.01), and ***(*P* < 0.001) indicate significant differences among groups. Numbers inside the columns of **(b)** and **(d**) indicate genotype and haplotype frequencies. **Figure S1. SNP1 =***BTA-77077-no-rs*; **SNP2 =***ARS-BFGL-NGS-112872*, **Figure S2. SNP1 =***ARS-BFGL-NGS-95538*; **SNP2 =***Hapmap47255-BTA-34035*; **SNP3 =***BTA-33950-no-rs*; **SNP4 =***Hapmap32551-BTA-128831;***SNP5 =***ARS-BFGL-NGS-14974*, **Figure S3. SNP1 =***Hapmap52325-rs29020544*; **SNP2 =***ARS-BFGL-NGS-57076*, **Figure S4. SNP1 =***Hapmap57515-ss46526957*; **SNP2 =***ARS-BFGL-NGS-117290*; **SNP3 =***UA-IFASA-5300*, and **Figure S5: SNP1 =***Hapmap47243-BTA-31267*. **Figure S6:** Genetic map of previously reported QTL for mastitis traits in Holstein populations and own results. On the right hand side of each chromosome, confidence intervals of previously reported QTL by linkage studies for SCS in red, SCC in green and clinical mastitis in blue are indicated (http://www.animalgenome.org/cgi-bin/QTLdb/BT/index); on the left hand side of each chromosome, arrows indicate the loci identified by GWAS for SCS in different Holstein populations; loci identified in our study are indicated with red arrows. Click here for file

Additional file 2: Table S1 SNPs associated with DYD for SCS stepwise adjustment for the most significant SNP. Chr = chromosome; and λ = genomic inflation factor (the inflation factor λ is the ratio of the observed median of the *χ*^2^ test statistic characterizing association between the phenotype and genetic markers and the expected median of this test statistic under the null hypothesis of no association predicted by theory (0.455 for 1df in association tests using the additive model)). Stepwise adjustment is based on adding the SNPs with the lowest *p*-value as a covariate, one by one. Different colors indicate significance level after Bonferroni correction at *p* ≤ 0.001 (green), ≤ 0.01 (yellow), and ≤ 0.05 (violet). Positions are according to the University of Maryland bovine genome assembly (UMD3.1). Click here for file
