# Supplementary figures and images for: Single nucleotide polymorphism and haplotype effects associated with somatic cell score in German Holstein cattle
Source: Genet Sel Evol. 2014 Jun 4;46(1):35. doi: 10.1186/1297-9686-46-35 (PMC4078941; doi:10.1186/1297-9686-46-35)

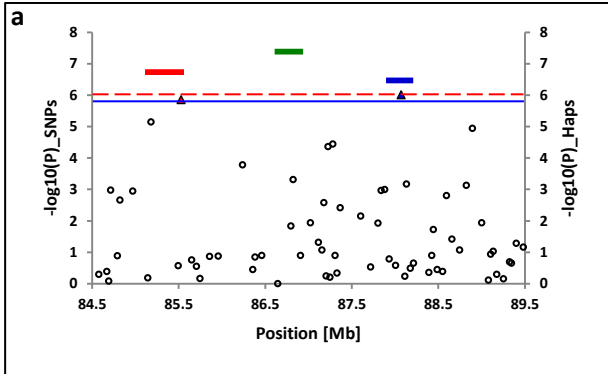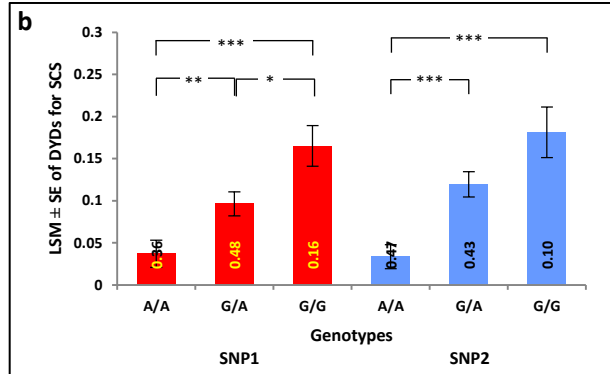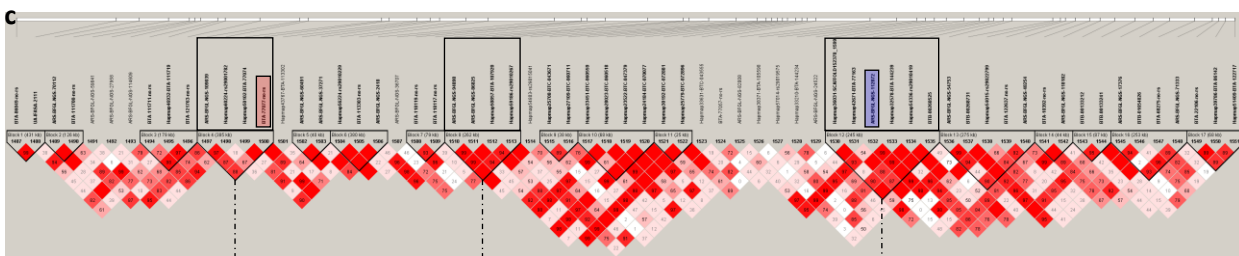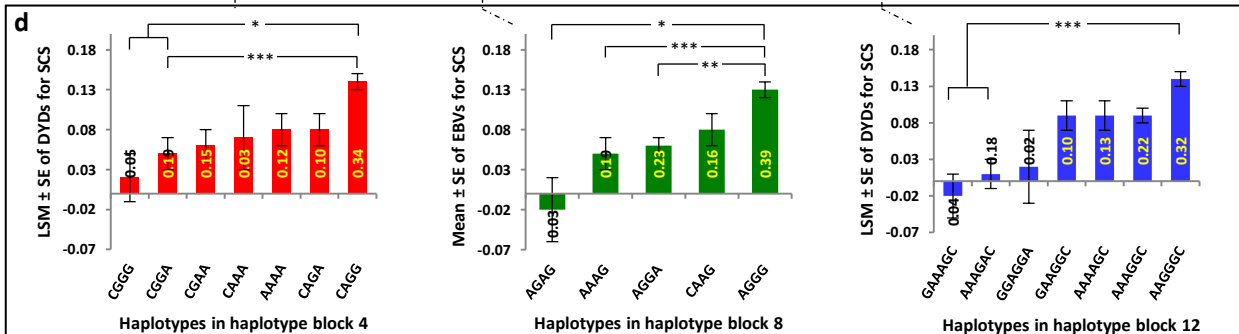

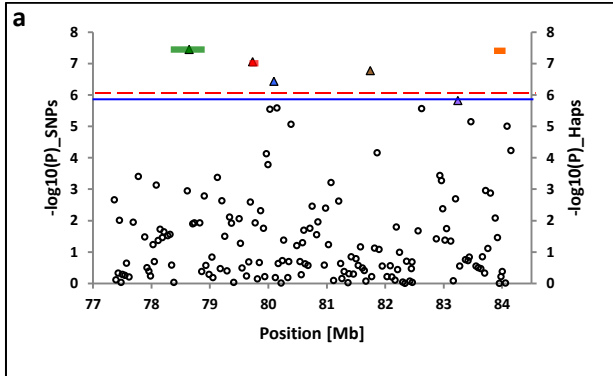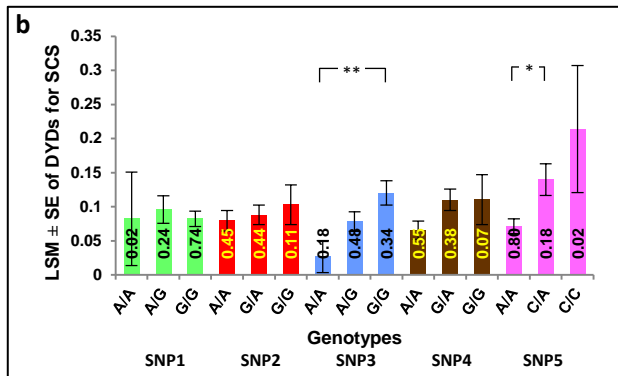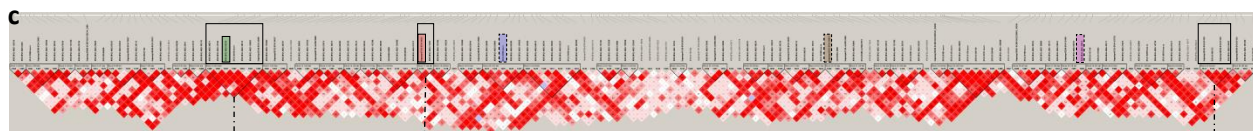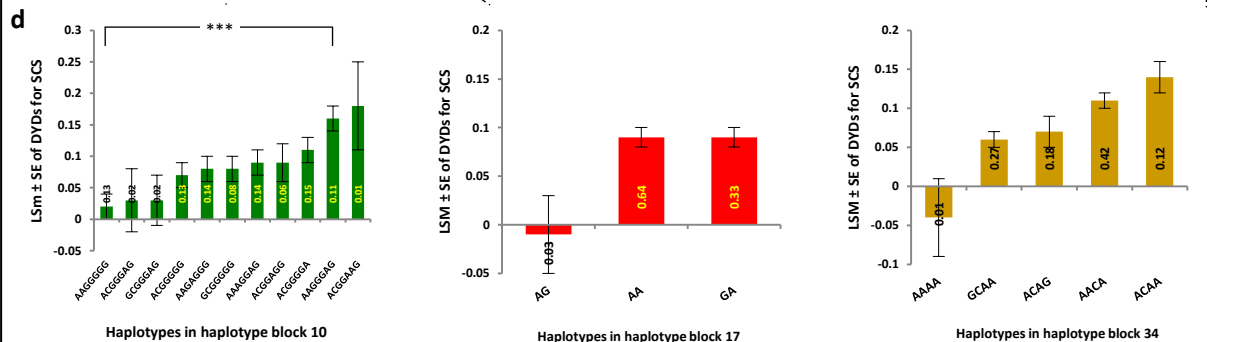

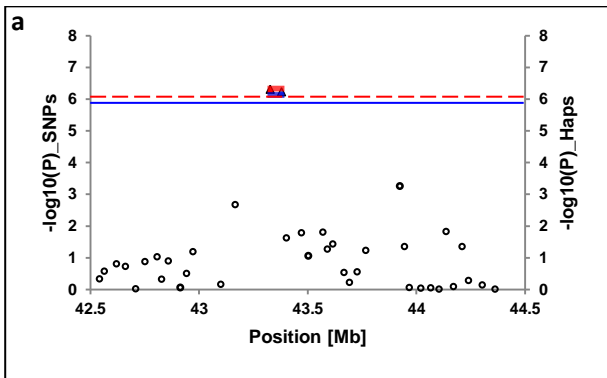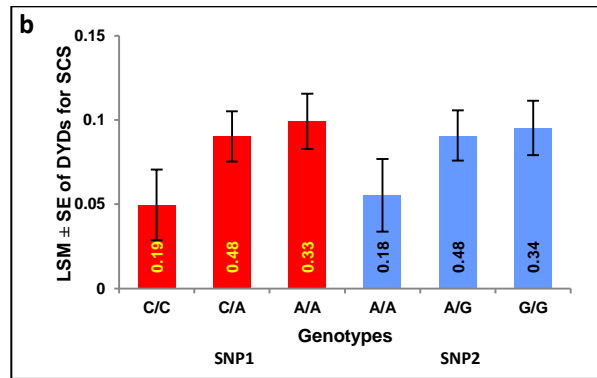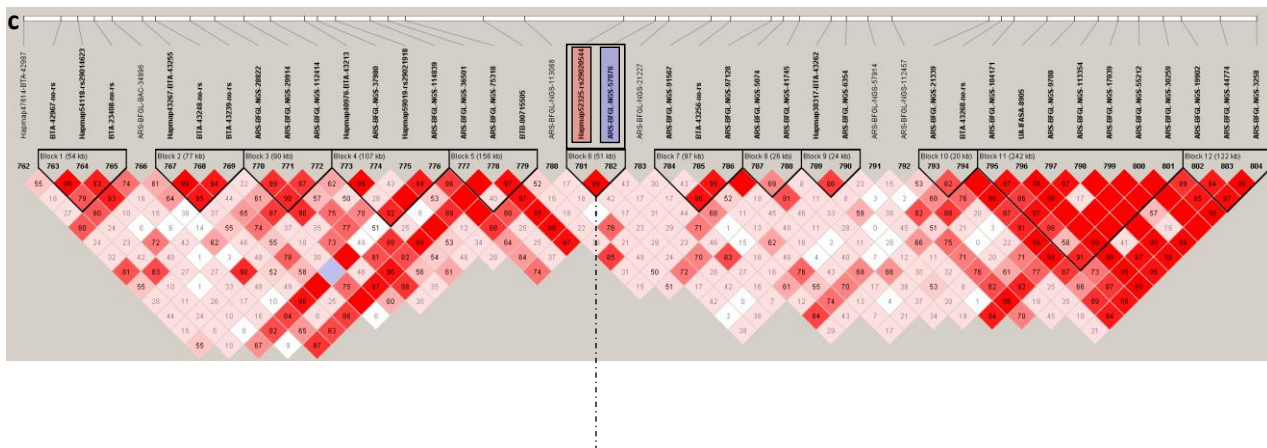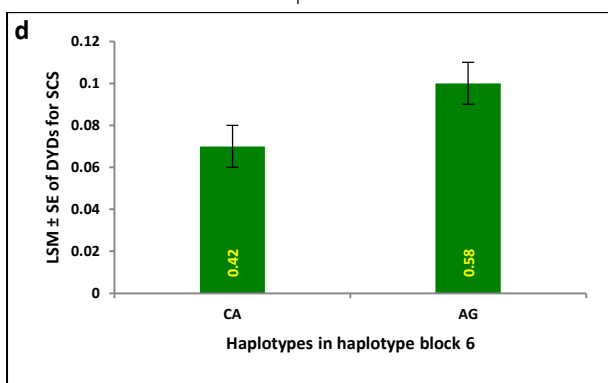

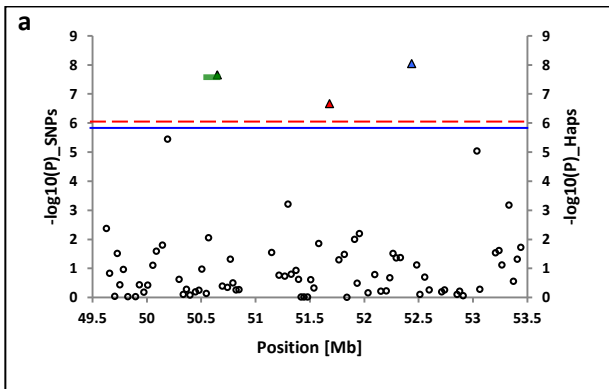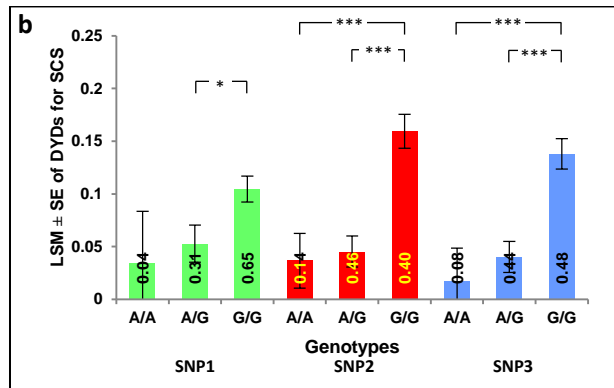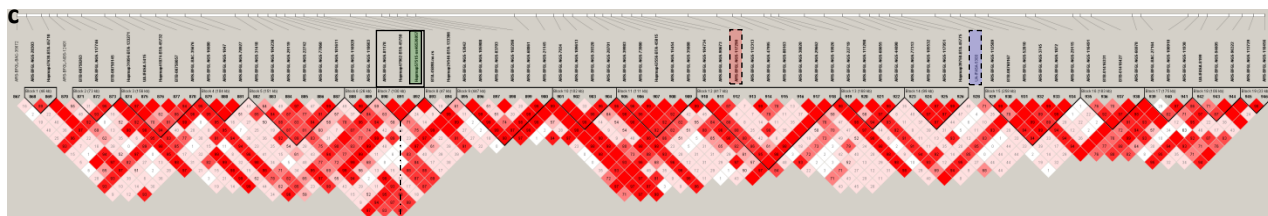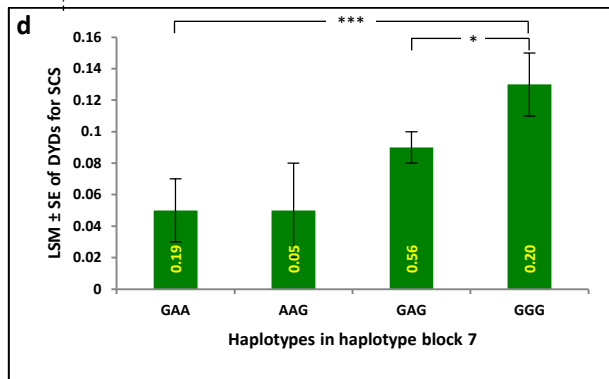

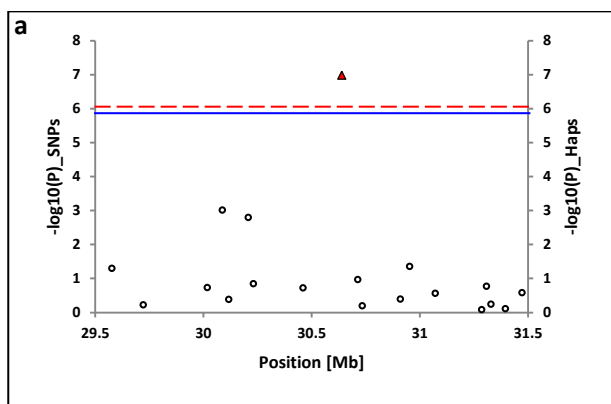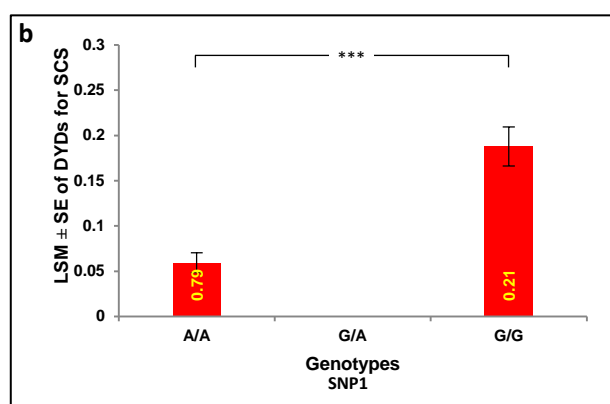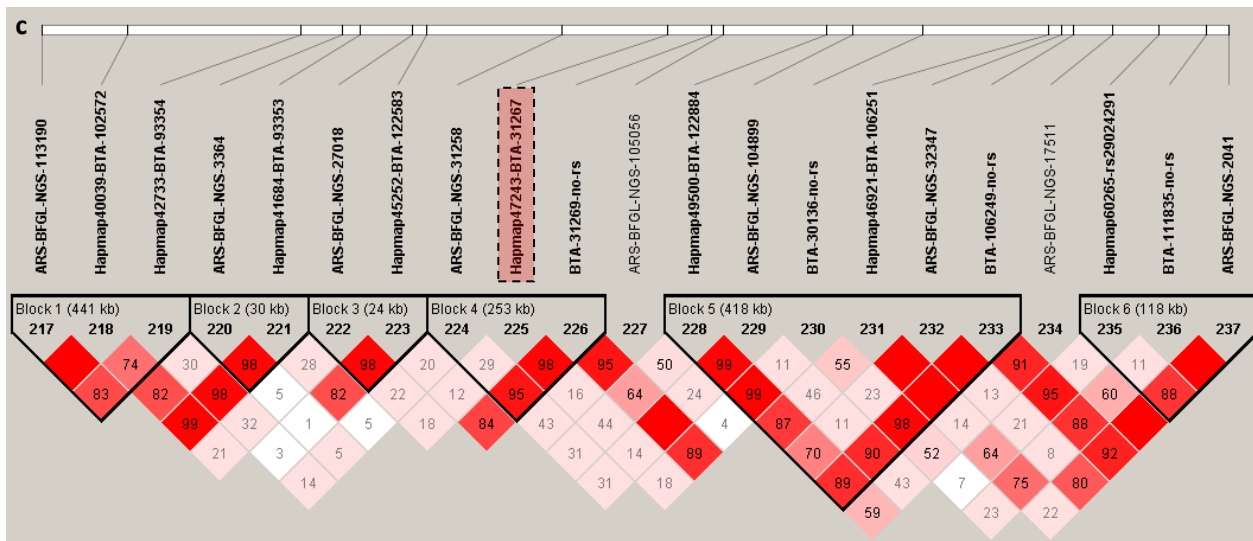

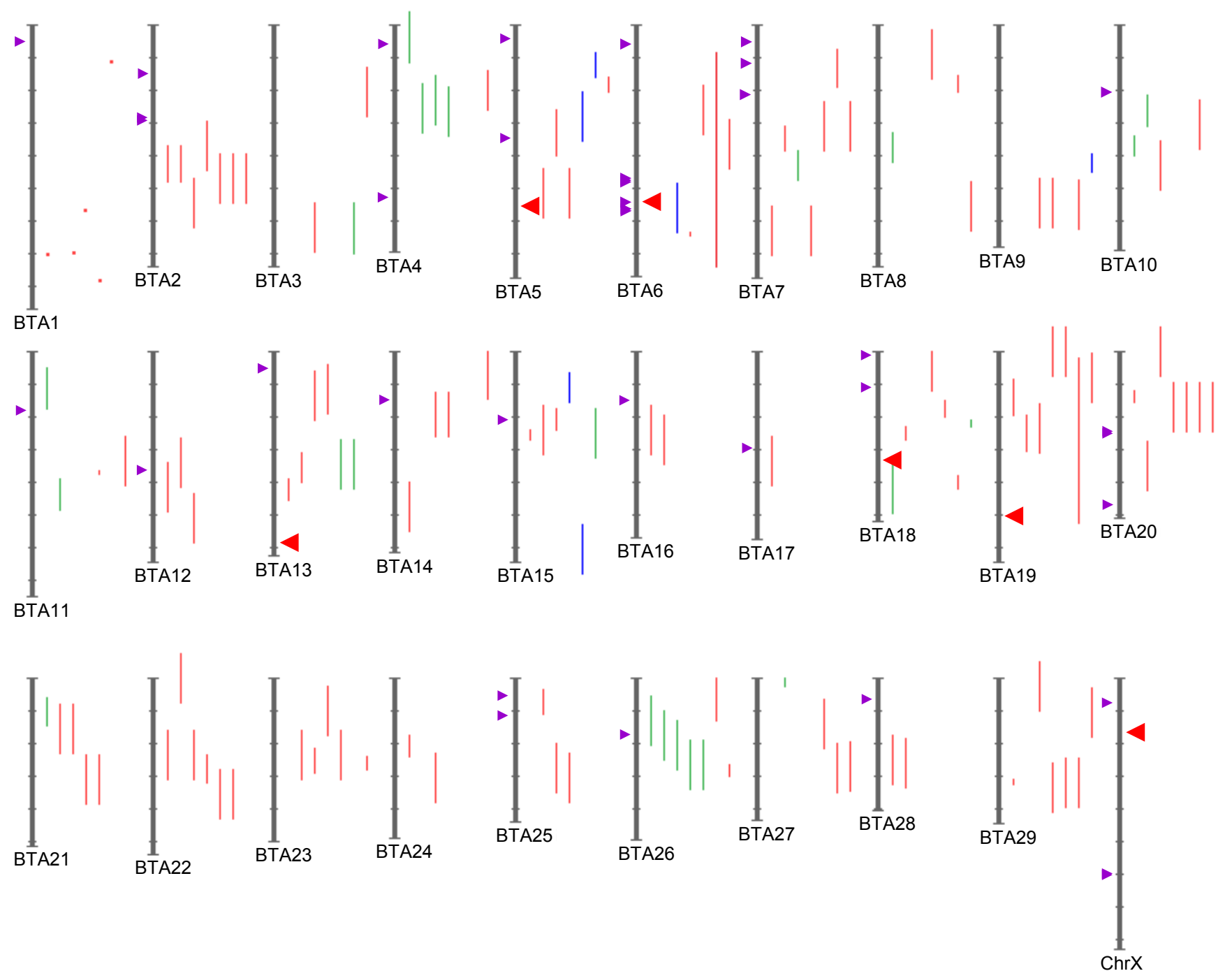

Supplement: Additional file 1: Figures S1, 2, 3, 4, and 5 — Significant regions on BTA6, 13, 18, 19 and X associated with DYD for SCS, respectively. (a) Manhattan plots for GWAS of significant SNPs and haplotypes; horizontal blue and red dashed lines indicate whole-genome significance thresholds at P ≤ 0.05 after Bonferroni correction for single markers and haplotypes, respectively; triangles refer to significant SNPs and bars refer to significant haplotypes. (b) Genotype effect plots of the significantly associated SNPs. (c) LD and haplotype block structure of the significant regions; each box represents the D′ values corresponding to each pair-wise SNP; haplotype blocks are indicated with black triangles, significant SNPs are highlighted in color and significant haplotypes are framed. (d) Haplotype effect plots of significantly associated haplotypes; *(P < 0.05), **(P < 0.01), and ***(P < 0.001) indicate significant differences among groups. Numbers inside the columns of (b) and (d) indicate genotype and haplotype frequencies. Figure S1. SNP1 =BTA-77077-no-rs; SNP2 =ARS-BFGL-NGS-112872, Figure S2. SNP1 =ARS-BFGL-NGS-95538; SNP2 =Hapmap47255-BTA-34035; SNP3 =BTA-33950-no-rs; SNP4 =Hapmap32551-BTA-128831;SNP5 =ARS-BFGL-NGS-14974, Figure S3. SNP1 =Hapmap52325-rs29020544; SNP2 =ARS-BFGL-NGS-57076, Figure S4. SNP1 =Hapmap57515-ss46526957; SNP2 =ARS-BFGL-NGS-117290; SNP3 =UA-IFASA-5300, and Figure S5: SNP1 =Hapmap47243-BTA-31267. Figure S6: Genetic map of previously reported QTL for mastitis traits in Holstein populations and own results. On the right hand side of each chromosome, confidence intervals of previously reported QTL by linkage studies for SCS in red, SCC in green and clinical mastitis in blue are indicated (http://www.animalgenome.org/cgi-bin/QTLdb/BT/index); on the left hand side of each chromosome, arrows indicate the loci identified by GWAS for SCS in different Holstein populations; loci identified in our study are indicated with red arrows. [file 1297-9686-46-35-S1.pdf]
